# Meat consumption reduction in Italian regions: Health co-benefits and decreases in GHG emissions

**DOI:** 10.1371/journal.pone.0182960

**Published:** 2017-08-15

**Authors:** Sara Farchi, Manuela De Sario, Enrica Lapucci, Marina Davoli, Paola Michelozzi

**Affiliations:** Department of Epidemiology of Regional Health Service, ASL Roma 1, Lazio Region, Rome, Italy; Universiti Putra Malaysia, MALAYSIA

## Abstract

**Introduction:**

Animal agriculture has exponentially grown in recent decades in response to the rise in global demand for meat, even in countries like Italy that traditionally eat a Mediterranean, plant-based diet. Globalization related dietary changes are contributing to the epidemic of non-communicable diseases and to the global climate crisis, and are associated with huge carbon and water footprints.

The objective of the study is to assess inequalities in health impacts and in attributable greenhouse gases-GHG emissions in Italy by hypothesizing different scenarios of reduction in red and processed meat consumption towards healthier consumption patterns more compliant with the recommendations of the Mediterranean food pyramid.

**Methods:**

We used demographic and food consumption patterns from national surveys and risk relationships between meat intake and cardiovascular and colorectal cancer mortality from IARC and other meta-analyses.

From the baseline data (year 2005–2006, average 406 gr/week beef and 245 gr/week processed meat), we considered hypothetical meat reduction scenarios according to international dietary guidelines such as the Mediterranean pyramid targets. For each geographical area (Northwest, Northeast, Centre, and South) and gender, we calculated the number of avoidable deaths from colorectal cancer, and cardiovascular disease among the adult population. Moreover, years of life gained by the adult population from 2012 to 2030 and changes in life expectancy of the 2012 birth cohort were quantified using gender-specific life tables.

GHG emission reductions under Mediterranean scenario were estimated only for beef by applying the Global Warming Potential (GWP) coefficient to total consumption and to a low carbon food substitution in adult diet.

**Results:**

The deaths avoidable (as percentage change compared to baseline) according to the three reduction scenarios for beef consumption were between 2.3% and 4.5% for colorectal cancer, and between 2.1% and 4.0% for cardiovascular disease; higher benefits would be observed in Northwestern areas and among males. In parallel, 5% and 6.4% of colorectal cancer and CVD deaths would be avoided if the Italian population ate the advised quantity of processed meat. Life table analysis suggests that the scenario that is fully compliant with the Mediterranean diet model would save 5 million years of life lost prematurely among men and women over the next 18 years and would increase average life expectancy of future generations by over 7 months.

Considering the environmental impact, emissions associated with the actual total intake of beef range from 12,900 to 21,800 Gg CO_2_ eq; emissions saved according to the Mediterranean scenario are in the range 8000–14000 Gg CO_2_ eq per year. The per capita reduction is 263 KgCO2eq/year/person with higher reductions in Northwestern and Central areas.

**Conclusions:**

In Italy, scenarios for reducing beef consumption are consistent with significant health and environmental co-benefits on current and future generations. Results support introducing policies to promote healthier behavior towards red and processed meat in the adult population within an overall balanced and healthy dietary pattern. Interventions should address gender, vulnerable population groups, and geographical differences in order to be more effective.

## Introduction

Climate change, land and water availability and biodiversity represent the most important challenges of the twenty-first century and involve identifying mitigation policies that have both immediate and long-term health benefits. Agriculture is now a dominant force behind many of these environmental threats [1]. Global livestock production in the world has increased substantially since the 1960s and beef production has more than doubled, due to a growing demand associated with human population growth, income growth and urbanization and the consequent fast lifestyles changes in diet and physical activity [2, 3]. Diets in industrialized countries, and even in countries with food patterns typical of a traditional Mediterranean diet like Italy, are characterized by high consumption of animal products and, therefore, high intake of saturated fat, above the recommendations of the World Health Organization (WHO) and World Cancer Research Fund of 300–400 grams of red meat per week (including beef, veal, pork, lamb, goat, horse) [4, 5].

The global food system is one of the major sources of greenhouse gas emissions (GHG). It is estimated that the agricultural sector contributes about one-fifth of total emissions and, of these about 80% are attributable to livestock [6, 7]. Of all meats, beef has the greatest impact on emissions, through production processes (CO_2_), the fermentation of ruminants (CH_4_), the cultivation of fodder and the use of fertilizers (NO) [8]. It has become clear that meat production also significantly contributes to the water footprint (WF), water pollution and water scarcity. The WF of any animal product is larger–in some cases 20 times than that of the WF of a crop with the same nutritional value. [9]

A diet rich in animal fat and other lifestyle risk factors such as physical inactivity, tobacco use and alcohol consumption have been estimated to account for 80% of type 2 diabetes, coronary heart disease, stroke and for one-third of cancers. Without preventative measures, the number of deaths from non-communicable diseases will increase by 17% globally over the next ten years according to estimates by the WHO [10]. A growing body of evidence shows the association between excess meat consumption, particularly of red and processed meat, and increased risk of premature death, in particular from heart disease, stroke, type 2 diabetes [11,12], and certain cancers [13,14]. Recently, IARC classified the consumption of red meat as probably carcinogenic to humans and the consumption of processed meat as carcinogenic to humans based on an evaluation of the evidence of colorectal cancer [15]. Recent systematic reviews shows also a higher risk of obesity among those who eat large amounts of red and processed meat.[16] Although the risks are not very high, the impact is huge since the exposed population, i.e. subjects who eat meat, is large and becoming even greater in high income countries and, at a faster rate, in emerging economies. According to the Global Burden of Disease Study [17], the risk factors related to diet contribute 10% to the entire global burden of disease.

The transition towards a western diet over the last decades has delayed mitigation solutions in the food sector in many countries [18]. In 2009, The Lancet included for the first time the reduction of animal products consumption (i.e. red meat, dairy products) among policies to reduce GHG emissions associated with significant health co-benefits. In comparative scenarios in UK and Brazil, a 30% reduction in livestock production resulted in a 15% health gain on cardiovascular-related disability-adjusted life years (DALY) [18]. A global impact evaluation provided evidence of even greater reductions up to 10% in total mortality by 2050 due mostly to the reduction in red meat consumption [19]. These co-benefits were associated with huge GHG reductions by 2050 (around 80–90%) [18,19]. Some studies suggest even small changes in meat consumption can have a measurable environmental benefit both in terms of GHG emissions and water footprint [20, 21]. A recent study, which used data on food consumption in Great Britain, suggested modifying the quantities of individual foods in the diet, according to nutritional recommendations of the WHO and acceptability by the English population, and consequent scenarios of reduced GHG emissions. The authors showed that even a modest modification in diet, with a reduction in meat consumption following nutritional guidelines, can lead to a reduction in the order of 20% of GHG emissions produced by the agriculture industry [20]. Moreover, other recent studies have estimated the impact of a shift towards recommended quantity of meat on WF, with reductions ranging from 23% to 41% of WF in southern and Western Europe [21].

No health or environmental impact evaluation has been carried out in countries like Italy where the traditional Mediterranean diet is followed and the potential burden in terms of diet-related deaths is expected to be relevant. In Italy, 13.5% of DALY are attributable to diet, making it the leading cause of premature mortality and disability [22].

In Italy, per capita meat consumption has tripled in the last 50 years with significant changes from the traditional Mediterranean dietary patterns, characterized by unprocessed and natural foods and a healthy profile of fat intake deriving mostly from vegetables and legumes rather than on meat [23]. The most accurate survey of food consumption by Italians refers to the years 2005–2006 (Survey INRAN) and indicates a profound transformation in eating habits, compared to the dietary patterns in the mid-nineties, with a progressive increase of meat consumption relative to overall dietary intake, towards that of the northern European countries. This is due to changes in lifestyle, the availability of a wide range of products, and sociodemographic changes [24]. In the sample, average fruit and vegetable consumption was just above the minimum recommended, while consumption of meat was greater than recommended [4]. Compared with European countries, Italian consumers rank medium-high in terms of red meat, in particular beef, and processed meat consumption [25].

This dietary transition has geographical differences, with greater intake of red meat and saturated fatty acids mainly in the northern regions of the country [24]. These underlying differences reflect different degrees of “penetration” of western lifestyle and may depend on urbanization levels, socio-economic factors, and traditional food habits possibly influenced by local agricultural production patterns, because most Italian livestock are raised in these regions [26]. The varying westernization is possibly related to the cultural safeguard of traditions that are stronger in certain areas of Italy than in others, especially in rural areas where 30% of Italian citizens live. One possible explanation of this cultural heritage is due to local products, with more than 250 products with a geographical indication logo, and culinary traditions, both varying across Italian regions, as do dietary habits. For example, processed meat accounts for almost 40 typical products with more than 90% of the factories located in the north of the country. Preserving this heritage represents an important defense against the negative aspects of globalization, not only since the traditional rural areas are more adherent to the healthy Mediterranean diet model [27], but also because their food habits prefer locally-produced food, lowering the environmental impact of the residents’ diets.

The objectives of this study are to evaluate the health and environmental impact in Italy of shifting habits of meat eaters (red and processed) towards healthier consumption patterns more compliant with the recommendations of the Mediterranean food pyramid [27]. Specifically, considering the heterogeneity of meat consumption attitudes, the aim is to investigate how gender and geographical differences contribute to inequalities in the impact of dietary changes.

## Data and methods

Starting from actual quantities consumed, we supposed three reduction scenarios of meat consumption by looking at beef and processed meat categories by gender and geographic area. According to the National Food Consumption Survey [24], “processed meat” is considered a unique food item while red meat comprises several meat items. Although the intuitive approach is to add up the amount of intake for each category, this approach would result in an over-estimate of consumption since it assumes that high-level consumers of one food are also high-level consumers of all of the others [25]. To overcome this problem, we used beef consumption as a proxy for all red meat.

### Meat consumption scenarios

To define the baseline scenario (the quantity of beef and processed meat consumed) we gathered the latest available data on average daily consumption by adult meat eaters (aged 18–64) by gender and geographical area from the National Food Consumption Survey (INRAN-SCAI) in 2005–2006 [24]. To define the scenarios (**S1 Table**), we referred to three different consumption targets. The first scenario, according to the World Cancer Research Fund advice [5], is compatible with the consumption of about 300g per person/week of red meat, which represents 40% of the total meat consumption in Italy. By applying this to the meat categories studied, the first scenario assumed a target of 244gr person/week for beef consumers and 147gr person/week for processed meat consumers. The second and third scenario are based on the Mediterranean Pyramid Model of recommended quantities [27] for red meat (150gr/week which represents 63% of the red meat currently consumed in Italy) and processed meat (not more than 50gr/week which represents 80% of processed meat currently consumed) respectively.

### Health benefits

We calculated the health impact due to reducing meat consumption in both annual chronic disease burdens and the long-term impact on overall mortality.

Concerning the first health outcome, for each geographical area and gender, we estimated the changes in cardiovascular disease (CVD) and colorectal cancer mortality that would occur if meat consumption were reduced from the baseline to the hypothetical scenarios. The assessment of avoidable deaths (AD) was ascertained using the following functions [28]:

$$\Delta_{AD}=y_{0}*\left(1-exp^{-\beta \Delta x}\right)*P_{0}$$

Which combine the dose-response association with mortality (β) and changes (i.e. difference) in consumption from baseline to suggested scenarios (ΔX) with data from adult meat-eaters (P_0_) and cause-specific mortality rate (y_0_).

The exposed population (P_0_) was estimated using data come from the National Statistics Office Multipurpose survey on families showing frequency of food consumption, by gender, age and geographical area over the period 2002–2012. In particular, referring to the most recent available year (2012), we defined the exposed population through the percentage of adults (aged 20+ years) that regularly at least one a week eats beef and processed meat. Similarly, we used data from the National Statistics Office survey on deaths and causes of death 2012 from cardiovascular diseases (CVD) and colorectal cancer, by gender, age and geographical area to define the baseline mortality rate (y_0_). Lastly, the coefficient β is based on a log-linear relationship between relative risk and meat consumption defined by epidemiological studies:

- **For red meat**: a dose–response relationship with colorectal cancer, with a 17% increased risk (95% CI 1.05–1.31) per 100 grams per day of red meat [15], and a 15% increased risk of mortality from CVD (95%CI 1.05–1.26) [12].
- **For processed meat**: 18% increase in risk of colorectal cancer (95% CI 1·10–1·28) per 50 grams per day [15], and a 24% risk of mortality from CVD (95%CI 1.09–1.40) [12].

In addition, 95% confidence intervals were calculated to evaluate the range of the variation of attributable deaths.

To estimate the long-term impact of healthier meat consumption habits we used life table methods based on the IOMLIFET model [29]. Separate life tables were constructed for the baseline scenario and the hypotheticals. We computed life tables distinctly for each geographical area and gender by populating them with the population of 2012 with age and sex-specific mortality rates for that year. We used a time-horizon of 106 years to predict gains in the 2012 newborn cohort life expectancy, while we emphasized the impact over the first 30 years (2030) for the 2012 population. The Mediterranean scenario was computed by applying the relative risk of overall mortality and meat consumption based on a meta-analysis of cohort studies:

**For red meat consumption**: RR = 1.21 (95%CI 1.15–1.26) for men and RR = 1.14 (95%CI 1.00–1.30) for women; **For processed meat consumption** RR = 1.23 (95%CI 1.10–1.37) for men and RR = 1.34 (95%CI 1.09–1.66) for women [12].

Based on these estimates, we calculated the decrease of age-specific mortality rates (aged 20 and above) corresponding to the difference in meat consumption between baseline and Mediterranean pyramid model scenarios.

### Environmental benefits

Owing to the high contribution of livestock ruminants to GHG emissions [7], evaluating the environmental impact focused only on beef consumption by comparing the baseline and the Mediterranean scenario.

We explored the potential beef contribution to GHG emissions attributable to total consumption (grams), consumer’s choices (energy intake) and dietary patterns (low-carbon food substitutions).

All the analyses estimated GHG emissions by coupling the mass and type of food with the specific Global Warming Potential (GWP) coefficient. We used GWP coefficients from life cycle assessment studies (LCAs) [7, 30, 31] since they allow calculating the environmental impact of a product throughout its lifecycle [32].

With respect to the assessment based on total intake, we estimated annual GHG emissions attributable to the total intake of beef in the diet of the adult meat-eating population. We provided two estimates. The first is based on the specific Italian livestock production chains (from cradle to farm gate), assuming that all the consumed meat was raised in Italy [30]. The second is based on a world average GWP coefficient that includes both the post-farm gate processes and the variability of beef production process among LCAs [7].

With respect to the assessment based on consumers’ choices, we evaluated the annual differences in GHG emissions from baseline to the Mediterranean scenario by varying the beef quality from maximum energy intake (low quality) to minimum energy intake (high quality) in the diet of adult meat-eaters [31]. These differences depend mostly on fat contents. The minimum and maximum energy intakes were retrieved from all the beef cuts regularly consumed in Italy [33] and measured as kcal/100 grams of edible portions. An edible portion corresponding to baseline and Mediterranean beef consumption was calculated using FAO correction coefficients [34].

Concerning dietary pattern assessment, we followed the approach of the Australian case study proposed by Clune and colleagues [7] that employed the GWP coefficient to estimate environmental impact for different sustainable diet substitution scenarios. We compared total GHG and food specific contributions for adult weekly per capita consumption at baseline and under the Mediterranean scenario with low-carbon food substitutions for beef. The beef substitute was proposed by the Italian Institute of Nutrition to preserve the overall nutritional balance [35]. We stratified this analysis by geographical area to account for the heterogeneity in the dietary patterns across Italy.

## Results

Table 1 presents average weekly meat consumption by the adult population and by regular beef and processed meat consumers. Average weekly meat consumption by the Italian population was 791 g, with clear geographical variations and differences by gender. Red meat is the most important component and beef by itself accounts for 40% of total meat consumption. Adult beef consumption per week was 406 grams, with large geographical and gender variations. Northwestern consumers had the highest intake (483gr/week) and also had the largest gender variation (546gr/week in males and 427gr/week in females), while southern consumers ate 343 grams of beef per week, and there was more homogeneity between the sexes. Processed meat consumption was less varied between areas, and ranged from 231gr/week in the Northwest to 259gr/week in the Northeast. Again, gender differences were more pronounced where consumption was higher (110gr/week more for males in the Northeast).

**Table 1:** Average weekly meat consumption by adults (total meat, beef and processed meat consumers), and reduction (%) to achieve Mediterranean target by gender and geographical area. Italy, 2005–2006.

Area/ gender	meat consumption (grams/week*)	Beef			Processed meat		
		*% consumers*^‡^	*grams/* *week consummed*	*Δ%* *(to 150 gr/week)*	*% consumers*	*grams/* *week consummed*	*Δ%* *(to 50 gr/week)*
NORTHWEST							
Males	910	*74*.*0*	546	*-72*.*5*	*69*.*2*	266	*-81*.*2*
Females	672	*66*.*9*	427	*-64*.*9*	*59*.*6*	203	*-75*.*4*
**Total**	**777**	***70*.*4***	**483**	***-68*.*9***	***64*.*4***	**231**	***-78*.*4***
NORTHEAST		* *		* *	* *		* *
Males	945	*66*.*2*	427	*-64*.*9*	*67*.*7*	315	*-84*.*1*
Females	644	*54*.*4*	350	*-57*.*1*	*55*.*4*	203	*-75*.*4*
**Total**	**784**	***60*.*3***	**385**	***-61*.*0***	***61*.*5***	**259**	***-80*.*7***
CENTRAL		* *		* *	* *		* *
Males	1022	*75*.*1*	455	*-67*.*0*	*67*.*4*	287	*-82*.*6*
Females	756	*70*.*9*	385	*-61*.*0*	*55*.*4*	210	*-76*.*2*
**Total**	**868**	***73*.*0***	**413**	***-63*.*7***	***61*.*3***	**245**	***-79*.*6***
SOUTH/ISLANDS		* *		* *	* *		* *
Males	868	*73*.*5*	378	*-60*.*3*	*70*.*9*	280	*-82*.*1*
Females	665	*68*.*6*	315	*-52*.*4*	*59*.*9*	203	*-75*.*4*
**Total**	**763**	***71*.*0***	**343**	***-56*.*3***	***65*.*3***	**245**	***-79*.*6***
ITALY		* *		* *	* *		* *
Males	917	*72*.*3*	448	*-66*.*5*	*69*.*2*	287	*-82*.*6*
Females	679	*65*.*7*	364	*-58*.*8*	*58*.*1*	203	*-75*.*4*
**Total**	**791**	***69*.*0***	**406**	***-63*.*1***	***63*.*6***	**245**	***-80*.*0***

*Inran-SCAI [24, 25]

^‡^*ISTAT [36]*

**Fig 1:**
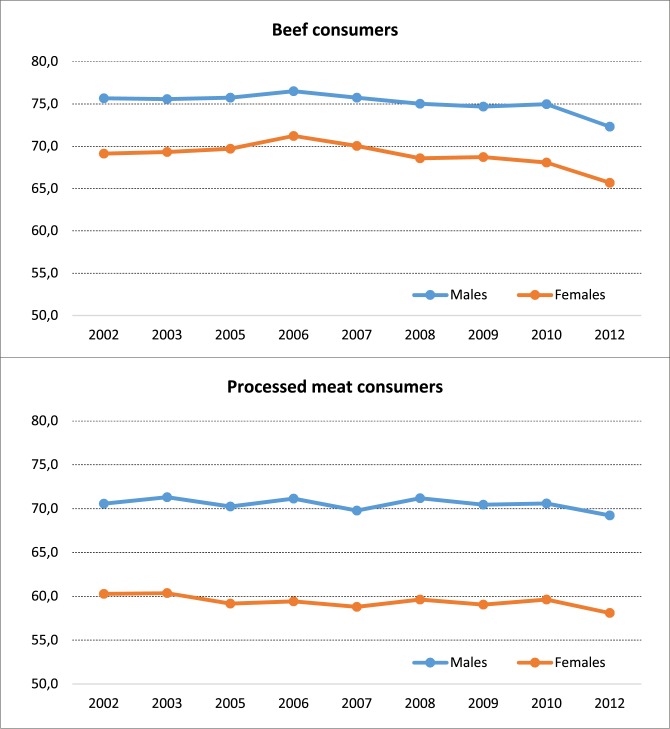
Time trend of regular beef and processed meat consumers, by gender. Italy, 2002–2012.

Time trends of bovine meat consumption show a steady decrease since 2006—stronger after 2010—in the proportion of regular beef eaters, while processed meat consumption time trend has been stable from 2002. (**Fig 1**).

**Fig 2:**
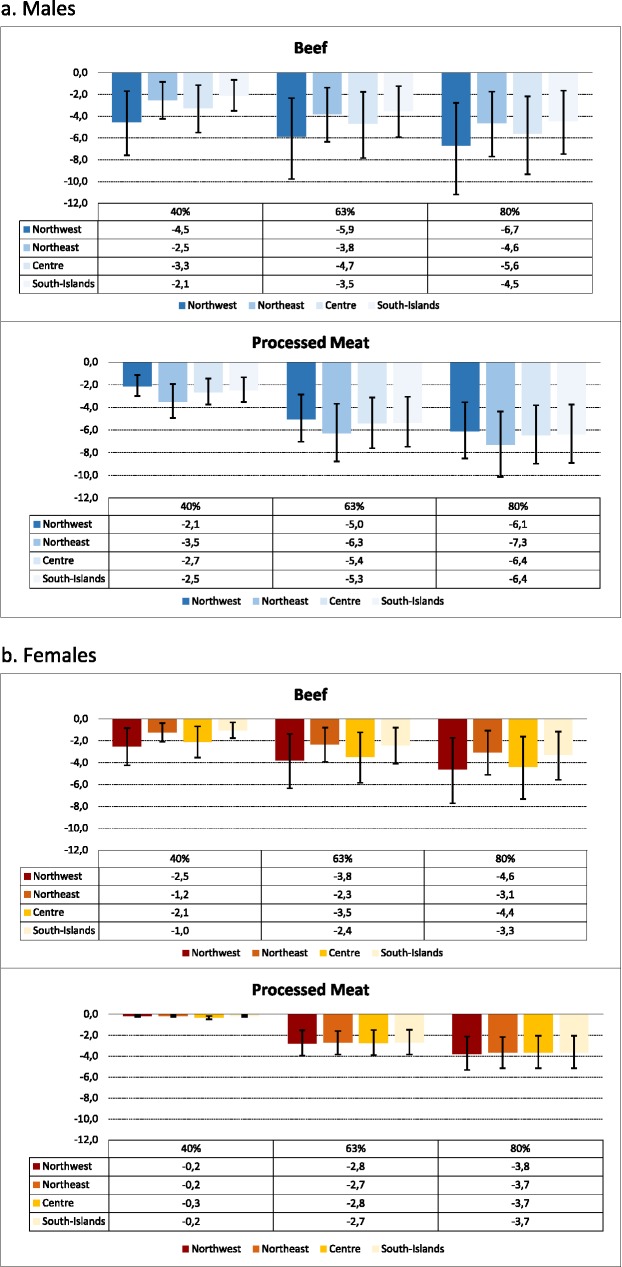
Percentage of avoidable deaths from colorectal cancer associated with different scenarios of reduction of beef and processed meat consumption (reduction: 40%, 63%, 80%) by gender and geographical area. **Italy 2012.** a. Males. b. Females.

Regarding the percentage of deaths avoidable, the more marked is the reduction of meat consumption, the greater are the health gains (**S2 Table**). If the Italian adult population would consume the recommended amount of beef, 3.7% and 3.3% of colorectal cancer and CVD deaths would be avoided. In parallel, 5% and 6.4% of colorectal cancer and CVD deaths would be avoided if the Italian population ate the advised quantity of processed meat. As expected, the health gains of decreasing beef consumption are reflected in the various consumption patterns of different population groups: higher for men and north westerners, less for women and southerners. There are clear gender and geographical differences in the benefits achieved (**Fig 2**).

Table 2 shows results of the IOMLIFET approach. Life table analysis suggests that if regular consumers reduced the amount of beef to 150 grams per week, globally over 30 years it would save 9 million of years of life lost (YOLL) prematurely, and if the intake of processed meat were 50 grams per week, almost 20 million YOLL would be saved. Differences between geographical areas and sexes are evident for beef, but less marked for processed meat.

**Table 2:** Life years gained by the Italian population over the period 2012–2030 given consumption of 150 grams per week of beef and 50 grams of processed meat.

Geographical area	Beef				Processed meat			
	Life years gained over 30 years (*thousand*)	Days per person	Life years gained over 30 years (thousand)	Days per person	Life years gained over 30 years (*thousand*)	Days per person	Life years gained over 30 years (*thousand*)	Days per person
	Males		Females		Males		Females	

**Northwest**	2231	107	1037	46	2627	125	2531	113
**Northeast**	1108	73	525	32	2264	148	1764	109
**Centre**	1261	82	652	39	2103	137	1956	118
**South-Islands**	1634	60	761	26	3516	128	3085	106
**Italy**	6234	79	2943	35	10631	135	9255	110

**Fig 3:**
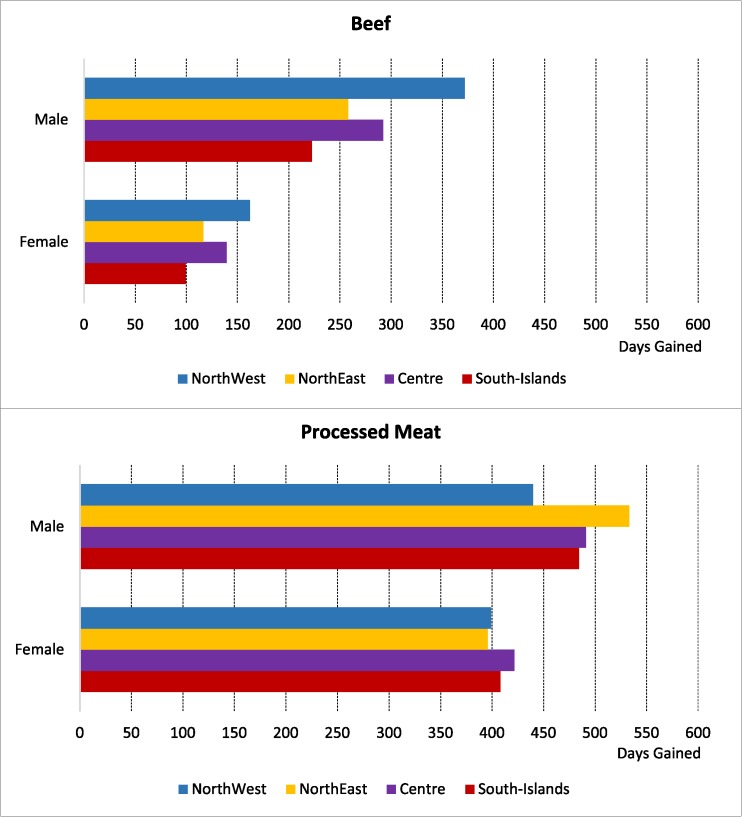
Change in life expectancy at birth (days gained) given consumption of 150 grams per week of beef and 50 grams of processed meat.

Considering the Italian birth cohort in 2012, an increase in average life expectancy of over 200 days for men and over 100 days for women would be observed if they decreased beef consumption, and over 400 days for men and women if they ate less processed meat. Again, gender and geographical differences are strong only for beef reduction in consumption (**Fig 3**).

Based on the specific Italian livestock’s production chain, a shift in beef consumption from the current 406 grams/week/person to the Mediterranean scenario (150 grams/week/person) could potentially save more than 8000 GgrCO2eq/year. This estimate ranges from 9713 up to 13776 GgrCO2eq/year when the analysis was based on world average GWP (**S3 Table**).

The results of GHG emissions related to energy intake of specific beef cuts show that in the baseline scenario the GHG could range from 5302 (low quality) to 3193 GgrCO2eq/year (high quality). The Mediterranean scenario coupled with the consumers’ choice of consuming better cuts of beef could save more than 4000 GgrCO2eq/year (**S4 Table**).

**Fig 4:**
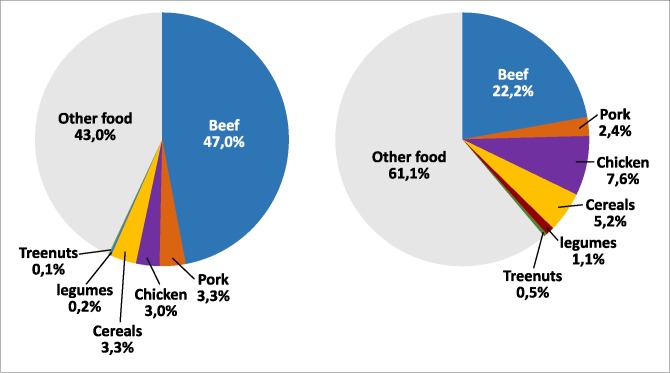
Total GHG and food contribution (%) associated with mean weekly per capita consumption by Italian adults at baseline and Mediterranean scenario.

Looking beyond only beef consumption—a full dietary choice at baseline is associated with 888.6 kgCo2eq/person/year with beef accounting for 47% of GHG emissions. A shift to the Mediterranean scenario with low-carbon food substitutions reduces total dietary GHG emissions (625.6 kgCo2eq/person/year) by redistributing the GHG contribution of each food (**Fig 4**).

This analysis highlights geographical differences in dietary patterns of other foods. In particular, the center and south of Italy have the highest consumption of pork (119g/week/person) while chicken consumption ranges from 119 in the Northwest to 168 in the Centre. The Northwest has the lowest consumption of pork (59 grams/week/person) and grains (below 1000 grams/week/person). Legumes consumption in all areas is below that needed for substitution quantity (see **S5 Table**).

The low-carbon food substitution scenario shows the greatest GHG reduction in Northwestern and Central areas of Italy. However, this reduction comes from different dietary changes. In Central Italy, it derived mostly from reducing in red meat consumption (beef and pork), and slightly increasing chicken consumption. In the Northwest on the contrary, the emission reduction derived from both eating less beef and making more low-carbon substitutions (mainly chicken, grains and legumes). The emission reduction was 244 Kg CO_2_ /year/person in the North- east and even lower in south (193 Kg CO_2_ /year/person).

(**Fig 5**).

**Fig 5:**
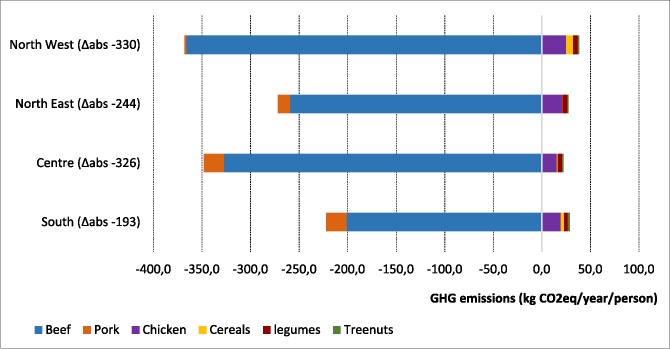
Differences in GHG emissions under the Mediterranean scenario with low carbon food substitutions, by Italian regions.

## Discussion and conclusions

### Health co-benefits

This study is the first to provide evidence of health and environmental (in terms of GHG emissions) benefits deriving from reducing red and processed meat consumption by the Italian adult population to the levels recommended by international and national healthy diet guidelines. Our study estimates the health impact of eating less red and processed meat in Italy. It shows that a relevant proportion of colorectal cancer and CVD deaths are attributable to meat intake. It also demonstrates that a change in dietary habits in Italy, coherent with the national guidelines, would bring benefits in term of life expectancy.

We chose the Mediterranean pyramid model as a reference since we assumed it was better suited for a Mediterranean country like Italy [27]. If Italian meat consumers would follow the Mediterranean pyramid model [27], which indicates consuming a maximum of 150 grams of beef per week, it would result in a 1.2% to 6.0% reduction in mortality from colorectal cancer and between 1.2% and 5.2% from CVD. In parallel, eating 50 grams of processed meat per week would correspond to between 3.0% and 7.2% fewer deaths from colorectal cancer and 2.7%-9.3% from CVD. Considering total mortality, the reductions proposed are able to improve life expectancy in a 2012 birth cohort by 4–9 months by 2030.

The Mediterranean targets are coherent with the recommended consumption limits recognized to be safe for people of all ages [37, 38]. Recommendations regarding red meat are guided by the principle of limiting saturated fats, which have a detrimental effect on total and LDL cholesterol by increasing the risk of cardiovascular disease [39].

Comparing these results with those from other countries, especially those from Great Britain [18, 20, 40], where most of the studies have been carried out, is not immediately possible, since reduction scenarios and the health outcomes considered are often different. Nevertheless, the results are consistent with those summarized in a 2013 review, which reported the burden of disease went down by between 1 and 16% [41].

Previous studies provided reductions in total mortality in the range of 0–18% [31], or 6–10% [19, 40] associated with global livestock or red meat reduction consumption. Other studies have evaluated the impact of hypothetical change in specific nutritional factors, such as saturated fats intake, leading to reductions in cardiovascular burden in the range of 6–10% in 9 European countries [42] and of 14% by 2025 in Sweden [43].

It is important to stress that in studies based on food frequency questionnaires, a possible misclassification of food consumption may occur that can result underestimate risk: for example, in the EPIC study the estimated risk for colon cancer associated with 100 grams increases of meat consumption jumps from 1.25 to 1.55 due to systematic and random dietary intake measurement errors [44].

It is therefore possible, given that we used food frequency data surveys to estimate meat consumption, that the results of the present study are underestimated and that the health gains could be even higher. It should be highlighted also that eating red meat is a risk factor for other diseases, such as stomach cancer, diabetes and obesity, diseases that were not taken into consideration in this analysis. It has also been postulated that reducing meat consumption would result also in improved dietary choices, for example an increase in fruit and vegetable intake, allowing further health benefits [19]. In the present study, health impacts were calculated based on the relative risks estimated in meta-analysis studies of meat consumption.

Nevertheless, the life table approach allows to estimate the impact on all-cause mortality and has been acknowledged to be especially suited to express effects of long-term exposures such as diet [45]. This method is beneficial since it is able to provide a clear estimate of the public health significance of proposed reductions in the consumption of red and processed meat.

This analysis considered only adults, because proposed reductions in red and cured meats are applicable only to the healthy adult population in whom we observed significant health benefits such as reducing cardiovascular and cancer risks. Results cannot be transferred to susceptible subgroups, such as children, women of childbearing age, or the elderly who may be more vulnerable to iron or zinc deficiencies [46].

### Geographical and gender differences

Few studies have evaluated the impact of geographical differences within the country of reduction scenarios of meat consumption, considering that health and environmental gains differed depending on variations in food production and consumption patterns [18, 19]. Our study showed potential health benefits deriving from the proposed dietary changes, which could be greater in northern areas where people tend to eat more. Such heterogeneity is consistent through the different analytical approaches we used (cause-specific mortality reductions in 2012 according to counterfactual scenarios, life years gained by Italian adults to 2030, improvement in life expectancy in future generations). Across Italian areas, there are slightly different culinary traditions and typical foods, but also differences in demographic and socioeconomic determinants that may affect attitudes towards a specific food. Processed meat consumption is uniform throughout Italy. There is a strong tradition of processed meat production, which includes ham, salami and other varieties. A survey conducted by the Italian Institute of Rural Sociology in 2002 counted more than 650 typical products from all Italian regions [47]. Nonetheless, this could be partly due to the higher costs of beef compared to processed meat, which makes consumption patterns easily influenced by social and economic conditions.

In all regions, there are fewer women who are regular consumers of red and processed meat, and they consume less than men do. Gender differences in specific food attitudes are well documented in western societies and possibly influenced by personal factors such as degree of health consciousness, and weight control that are more common in females than in males. In our study, Italian women not only eat less red meat but they also have a different baseline mortality risk for cardiovascular and colorectal cancer compared with men. These results underline the importance of developing gender-specific nutritional goals for the general population.

### GHG emission impact

Current beef consumption by Italian carnivores has an environmental impact in the range of 12,900 to 21,800 Gg CO2eq. per year of GHG emissions. Emission savings according to the Mediterranean pyramid model are between 8000 and 14000 Gg CO2eq. per year. The Mediterranean scenario with low-carbon food substitutions reliable for the Italian population [35] produced an overall per capita reduction of 263 kg CO2eq./year. These potentially avoidable emissions thanks to dietary change are not negligible since overall annual per capita emissions from all sources (not only agriculture) are estimated to be around 7 tons CO2 eq./year [48]. The Italian Institute of Nutrition study produced a net environmental benefit similar to ours (114,6 kg CO2 eq. per capita saved per year) but it did not analyse regional variations [35]. Our study suggests that assessing the environmental impact needs to account for geographical heterogeneity in dietary habits across Italy. The low-carbon food substitute highlights that the greatest GHG benefits can be obtained in the Northwestern and Central regions where baseline beef consumption is higher. Moreover, GHG reductions in the same amount were obtained from different dietary changes.

Other studies have provided similar estimates of environmental benefit like the GHG emissions in the range of 29–70% associated with red meat consumption reductions [19], or have evaluated overall dietary changes, i.e. shifting to a vegetarian diet with reductions between 22–29% [49] or a Mediterranean diet (GHG reductions in the range 10–30%) [50]. Scenarios for reducing beef consumption at Mediterranean targets in Italy seem not only in line with recommended nutritional levels but also address reduction goals of GHG, which the European Union agreed to in Paris in November, 2015 [51]. The Paris agreements anticipate, in fact, a reduction of at least 40% in domestic emissions by 2030 and 80% by 2050. Various studies have stressed that adopting a diet that conforms to the guidelines would contribute to a substantial reduction in GHG emissions associated with food consumption [3]. According to the FAO, meat consumption is expected to increase by about 73% by 2050 due to population and income increases, even in developing countries [23]. To balance this increase, policies to reduce consumption are essential in order to achieve tangible benefits on population health and the environment. We used two GWP coefficients associated with beef production activity, one from an Italian LCA study [30] and another from a published meta-analysis of LCA studies including post-farm emissions which provided comparable food-specific coefficients on which we based the substitution analysis [7]. We also used a third coefficient based on energy units, to account for different cuts of beef consumed [31]. Although we followed a methodology previously suggested by others [7], our estimates are a simplification, since they do not correct the amount of food produced for the quantity actually consumed, stored, cooked, and wasted in the Italian context.

### Future research avenues

Our results confirm significant health impacts of reducing red meat consumption to the Mediterranean target (150 grams/week/person) and suggest also important environmental gains. Although using scenarios is a simplification, it opens the way to studies investigating more in-depth alternatives to red meat in the dietary pattern, that can account not only for quantities and nutritional balance but also for costs, acceptability, and regional culinary traditions. Also, different age groups have different dietary needs and substitution patterns, making further study necessary.

### Policy implications

The main public health message from this study is that small changes in habits towards red and processed meat, towards higher quality and smaller quantities, can maximize health impacts and minimize the environmental burden in terms of GHG emissions. Due to the heterogeneous health and environmental impact, policy recommendations will need to target specific Italian areas. Overall, the reductions in red meat consumption we propose are compatible with a Mediterranean-type diet, which indicates a variety of proteins (meat and poultry, fish, milk and dairy products, legumes) and does not suggest radical choices, such as the adoption of a vegetarian or vegan diet [27]. For example, diets with fewer animal-sourced foods typically include more legumes, nuts and whole grains which evidence suggests have health benefits and are likely to increase the number of avoided deaths [31, 40, 42, 43].

Possible interventions to reduce emissions from livestock production are based on technologies and practices that improve production efficiency, but mitigation and adaptation options have to include public health policies to reduce meat consumption. In practice, people may be informed and invited to reduce portion size or frequency of consumption of red meat and processed meat. However, how to reach these goals is challenging and should take into account factors influencing food choice, and the external economic and social context in which choices are made. One possible solution is pricing food-related GHG emissions that have been proved to be effective in Australia [19]. Such policies need to address health inequalities by income since poorer people are more prone to make unhealthy choices and are more resistant to possible changes than richer subgroups. The Australian study partly addressed this issue by proposing the use of taxation of GHG emissions from red meat and other foods for health promotion in the poorest population subgroups [19]. Another information action is dietary guidelines to raise awareness of population of environmental impacts of the so-called “discretionary” food, emphasizing that following guidelines is good not only for one’s health but also for the environment [37]. Examples of interventions focusing on achieving a full healthy diet approach rather than on a single objective such as reducing red meat consumption are becoming widespread especially in the US, but also in Europe. These interventions promote healthy eating through educational interactive material (website, app for mobile phones). To address low income families, specific tools are used, such as meetings, support groups, and, more recently, food subsidy programmes (e.g. food vouchers) that have found to improve diet quality in the UK [52].

Dairy and beef fat typically contains around 3–6% TFA (wt. % of total fatty acids), while levels in lamb and mutton can be somewhat higher

The TFA content of fat from pork and poultry are generally below 1% of total fatty acids, but the content may vary, primarily depending on the TFA content of the feed (Aro et al., 1998a).

Among the specific efforts to be set up in this field, there is the need to raise awareness of the environmental impact of different kinds of food, to promote model food consumption which result in the lowest greenhouse gas emissions and sufficient, balanced amounts of protein and micronutrients, and which may be more sustainable.

Experts have highlighted that climate change mitigation policies are difficult to implement in part because their effects are not geographically limited, and also because they will have impact decades from now. Empowering both the general population and policy makers is then a crucial goal [41].

## Conclusions

This study provides, for the first time in Italy, evidence supporting a reduction in red meat consumption toward the Mediterranean target of 150 grams/week per capita. The expected health and environmental gains are huge, but heterogeneity across geographical areas suggests the importance of detailed investigation of local determinants of the observed variations. Strategies to empower citizens to make dietary changes could be improved in Italy by accounting for local differences. At the same time, the environmental consciousness of the general population needs to expand. Now and in the future public health will play an important role in prioritizing the resources and protecting the vulnerable groups most affected by unhealthy lifestyles and by environmental injustice.

## Supporting information

S1 Table Scenarios of reduction of beef and processed meat consumption among Italian adult population. (DOCX)

S2 Table Estimate of avoidable deaths (No. and %) from colorectal cancer and CVD associated with different scenarios of reduction of beef and processed meat consumption (reduction: 40%, 63%, 80%). Italy, 2012. (DOCX)

S3 Table Annual GHG emission for baseline and Mediterranean scenario based on mass unit GWP coefficient for adult Italian consumers. (DOCX)

S4 Table Annual GHG emission for baseline and Mediterranean scenario based on energy unit GWP coefficient for adult Italian consumers. (DOCX)

S5 Table Consumption of the main food groups and GHG profiles associated with the baseline and Mediterranean scenarios by Italian regions. (DOCX)
