# Supplementary material for: Meat consumption reduction in Italian regions: Health co-benefits and decreases in GHG emissions
Source: PLoS One. 2017 Aug 15;12(8):e0182960. doi: 10.1371/journal.pone.0182960 (PMC5557600; doi:10.1371/journal.pone.0182960)
Supplement: S1 Table — (DOCX) [file pone.0182960.s001.docx]

**Table S1 – Scenarios of Reduction of beef and processed meat consumption among Italian Adult Population**

| Reduction | Beef consumption  gr/week | Processed meat consumption gr/week |
| --- | --- | --- |
|  |  |  |
| *Baseline Scenario˟* | **406** | **245** |
| 40% | 244 | 147 |
| 63% | 150^‡^ | 91 |
| 80% | 81 | 49^‡^ |

˟ Sources: INRAN-SCAI 2005-2006

^‡^ Mediterranean Scenario
