# Supplementary material for: Meat consumption reduction in Italian regions: Health co-benefits and decreases in GHG emissions
Source: PLoS One. 2017 Aug 15;12(8):e0182960. doi: 10.1371/journal.pone.0182960 (PMC5557600; doi:10.1371/journal.pone.0182960)
Supplement: S2 Table — (DOCX) [file pone.0182960.s002.docx]

**Table S2 - Estimate of avoidable deaths (No. and %) from colorectal cancer and CVD associated with different scenarios of reduction of beef and processed meat consumption (reduction: 40%, 63%, 80%). Italy, 2012**
