# Supplementary material for: Meat consumption reduction in Italian regions: Health co-benefits and decreases in GHG emissions
Source: PLoS One. 2017 Aug 15;12(8):e0182960. doi: 10.1371/journal.pone.0182960 (PMC5557600; doi:10.1371/journal.pone.0182960)
Supplement: S3 Table — (DOCX) [file pone.0182960.s003.docx]

**Table S3 - Annual GHG Emission for Baseline and Mediterranean Scenario based on mass unit GWP coefficient for adult Italian consumers**

|  | | | | |
| --- | --- | --- | --- | --- |
| Beef data | Italian LCA ^1^ | | World Average LCA ^2^: Median (Q1;Q3) | |
|  | Baseline | Mediterranean | Baseline | Mediterranean |
| Consumers | 32,689,645 | 32,689,645 | 32,689,645 | 32,689,645 |
| Consumption gr/week/person | 406 | 150 | 406 | 150 |
| GWP | 18.7 kg CO2-eq /kg Beef | | 26.61 (22.26 ; 31.57) kg CO2-eq /kg Bone Free Meat | |
| **Annual GHG emission Gg CO2 eq** | **12941** | **4781** | **18415**  (15405 ; 21848) | **6804**  (5691 ; 8072) |
| ***Difference ( Gg CO2 eq)*** | ***-8160*** | | ***-11612*** *(-9713 ; -13776)* | |

*^1^ Coderoni S, Valli L, Pignedoli S, Tozzi L, Pantano A, Vinci A. Greenhouse gases emissions from italian livestocks. Which scenarios? Ministry of Forestry and Agricolture, National Institute for Agrarian Economy. 2014. [in Italian]*

*^2^ Clune S, Crossin E, Verghese K. Systematic review of greenhouse gas emissions for different fresh food categories. J Clean Prod. 2017; 140: 766-783. doi: 10.1016/j.jclepro.2016.04.082.*
