# Supplementary material for: Meat consumption reduction in Italian regions: Health co-benefits and decreases in GHG emissions
Source: PLoS One. 2017 Aug 15;12(8):e0182960. doi: 10.1371/journal.pone.0182960 (PMC5557600; doi:10.1371/journal.pone.0182960)
Supplement: S4 Table — (DOCX) [file pone.0182960.s004.docx]

**Table S4 - Annual GHG Emission for Baseline and Mediterranean Scenario based on energy unit GWP coefficient for adult Italian consumers**

|  | | | | |
| --- | --- | --- | --- | --- |
| Beef | Baseline | | Mediterranean | |
|  | Low quality | High quality | Low quality | High quality |
| Consumers | 32,689,645 | 32,689,645 | 32,689,645 | 32,689,645 |
| Consumption gr/week/person p.e. | 324.8 | 324.8 | 120.0 | 120.0 |
| Energy intake  Kcal/100gr p.e | 171 | 103.0 | 171 | 103.0 |
| GWP ^1^ | 5.6 gr CO2-eq /Kcal | | 5.6 gr CO2-eq /Kcal | |
| **Annual GHG emission Gg CO2 eq** | **5302** | **3193** | **1959** | **1180** |
| ***Differences*** |  | |  | |
| From Baseline High Quality to Mediterranean Low Quality | | -1234 |  |  |
| From Baseline High Quality to Mediterranean High Quality | | -2013 |  |  |
| From Baseline Low Quality to Mediterranean low Quality | | -3343 |  |  |
| From Baseline Low Quality to Mediterranean High Quality | | -4122 |  |  |

*^1^* [*Tilman*](http://www.nature.com/nature/journal/v515/n7528/abs/nature13959.html#auth-1) *D,* [*Clark*](http://www.nature.com/nature/journal/v515/n7528/abs/nature13959.html#auth-2) *M. Global diets link environmental sustainability and human health. Nature. 2014;515: 518–522. doi:10.1038/nature13959*
