# Supplementary material for: Meat consumption reduction in Italian regions: Health co-benefits and decreases in GHG emissions
Source: PLoS One. 2017 Aug 15;12(8):e0182960. doi: 10.1371/journal.pone.0182960 (PMC5557600; doi:10.1371/journal.pone.0182960)
Supplement: S5 Table — (DOCX) [file pone.0182960.s005.docx]

**Table S5 – Consumption of the main food groups and GHG profiles associated with the Baseline and Mediterranean scenarios by Italian regions**

| **Italy** | GWP | Baseline Scenario | | | Mediterranean Scenario | | | ΔCO2 (kg/year/person) | |
| --- | --- | --- | --- | --- | --- | --- | --- | --- | --- |
|  |  | Consumption | GHG profile (Kg CO2/year/person) | | Consumption | GHG profile (Kg CO2/year/person) | |  |  |
|  | *Median (gr CO2 /gr product)* | gr/week/person | Abs | % | gr/week/person | Abs | % | Abs | % |
| **Beef** | **26.61** | **301** | **417.64** | **47.0** | **100** | **138.75** | **22.2** | **-278.9** | **-66.8** |
| **Pork** | **5.77** | **98** | **29.48** | **3.3** | **50** | **15.04** | **2.4** | **-14.4** | **-49.0** |
| **Chicken** | **3.65** | **140** | **26.65** | **3.0** | **250** | **47.58** | **7.6** | **20.9** | **78.6** |
| Eggs | 3.46 | 147 | 26.52 | 3.0 | 147 | 26.52 | 4.2 | - | - |
| Fish: | 3.49 | 322 | 58.60 | 6.6 | 322 | 58.60 | 9.4 | - | - |
| Milk | 1.29 | 721 | 48.50 | 5.5 | 721 | 48.50 | 7.8 | - | - |
| Cheese | 8.55 | 420 | 187.25 | 21.1 | 420 | 187.25 | 29.9 | - | - |
| **Cereals** | **0.5** | **1113** | **29.02** | **3.3** | **1240** | **32.33** | **5.2** | **3.3** | **11.4** |
| **legumes** | **0.51** | **77** | **2.05** | **0.2** | **250** | **6.65** | **1.1** | **4.6** | **224.7** |
| Vegetables | 0.37 | 1554 | 29.98 | 3.4 | 1554 | 29.98 | 4.8 | - | - |
| Fruits | 0.42 | 1442 | 31.58 | 3.6 | 1442 | 31.58 | 5.0 | - | - |
| **Treenuts** | **1.2** | **21** | **1.31** | **0.1** | **45** | **2.82** | **0.5** | **1.5** | **114.3** |
| **Total** |  | **6356** | **888.6** | **100** |  | **625.6** | **100** | **-263.0** | **-29.6** |

| **Northwest** | GWP | Baseline Scenario | | | Mediterranean Scenario | | | ΔCO2 (kg/year/person) | |
| --- | --- | --- | --- | --- | --- | --- | --- | --- | --- |
|  |  | Consumption | GHG profile (Kg CO2/year/person) | | Consumption | GHG profile (Kg CO2/year/person) | |  |  |
|  | *Median (gr CO2 /gr product)* | gr/week/person | Abs | % | gr/week/person | Abs | % | Abs | % |
| **Beef** | **26.61** | **364** | **9.69** | **1.1** | **100** | **2.66** | **0.4** | **-366.3** | **-72.5** |
| **Pork** | **5.77** | **56** | **0.32** | **0.0** | **50** | **0.29** | **0.0** | **-1.8** | **-10.7** |
| **Chicken** | **3.65** | **119** | **0.43** | **0.0** | **250** | **0.91** | **0.1** | **24.9** | **110.1** |
| Eggs | 3.46 | 133 | 0.46 | 0.1 | 133 | 0.46 | 0.1 | - | - |
| Fish: | 3.49 | 273 | 0.95 | 0.1 | 273 | 0.95 | 0.2 | - | - |
| Milk | 1.29 | 742 | 0.96 | 0.1 | 742 | 0.96 | 0.2 | - | - |
| Cheese | 8.55 | 420 | 3.59 | 0.4 | 420 | 3.59 | 0.6 | - | - |
| **Cereals** | **0.5** | **952** | **0.48** | **0.1** | **1240** | **0.62** | **0.1** | **7.5** | **30.3** |
| **legumes** | **0.51** | **77** | **0.04** | **0.0** | **250** | **0.13** | **0.0** | **4.6** | **224.7** |
| Vegetables | 0.37 | 1561 | 0.58 | 0.1 | 1561 | 0.58 | 0.1 | - | - |
| Fruits | 0.42 | 1575 | 0.66 | 0.1 | 1575 | 0.66 | 0.1 | - | - |
| **Treenuts** | **1.2** | **21** | **0.03** | **0.0** | **45** | **0.05** | **0.0** | **1.5** | **114.3** |
| **Total** |  | **6293** | **18.2** | **2** |  | **11.9** | **2** | **-329.6** | **-34.8** |

| **Northeast** | GWP | Baseline Scenario | | | Mediterranean Scenario | | | ΔCO2 (kg/year/person) | |
| --- | --- | --- | --- | --- | --- | --- | --- | --- | --- |
|  |  | Consumption | GHG profile (Kg CO2/year/person) | | Consumption | GHG profile (Kg CO2/year/person) | |  |  |
|  | *Median (gr CO2 /gr product)* | gr/week/person | Abs | % | gr/week/person | Abs | % | Abs | % |
| **Beef** | **26.61** | **287** | **7.64** | **0.9** | **100** | **2.66** | **0.4** | **-259.5** | **-65.2** |
| **Pork** | **5.77** | **91** | **0.53** | **0.1** | **50** | **0.29** | **0.0** | **-12.3** | **-45.1** |
| **Chicken** | **3.65** | **140** | **0.51** | **0.1** | **250** | **0.91** | **0.1** | **20.9** | **78.6** |
| Eggs | 3.46 | 147 | 0.51 | 0.1 | 147 | 0.51 | 0.1 | - | - |
| Fish: | 3.49 | 308 | 1.07 | 0.1 | 308 | 1.07 | 0.2 | - | - |
| Milk | 1.29 | 686 | 0.88 | 0.1 | 686 | 0.88 | 0.1 | - | - |
| Cheese | 8.55 | 413 | 3.53 | 0.4 | 413 | 3.53 | 0.6 | - | - |
| **Cereals** | **0.5** | **1232** | **0.62** | **0.1** | **1240** | **0.62** | **0.1** | **0.2** | **0.6** |
| **legumes** | **0.51** | **49** | **0.02** | **0.0** | **250** | **0.13** | **0.0** | **5.3** | **410.2** |
| Vegetables | 0.37 | 1673 | 0.62 | 0.1 | 1673 | 0.62 | 0.1 | - | - |
| Fruits | 0.42 | 1463 | 0.61 | 0.1 | 1463 | 0.61 | 0.1 | - | - |
| **Treenuts** | **1.2** | **28** | **0.03** | **0.0** | **45** | **0.05** | **0.0** | **1.1** | **60.7** |
| **Total** |  | **6517** | **16.6** | **2** |  | **11.9** | **2** | **-244.2** | **-28.3** |

| **Centre** | GWP | Baseline Scenario | | | Mediterranean Scenario | | | ΔCO2 (kg/year/person) | |
| --- | --- | --- | --- | --- | --- | --- | --- | --- | --- |
|  |  | Consumption | GHG profile (Kg CO2/year/person) | | Consumption | GHG profile (Kg CO2/year/person) | |  |  |
|  | *Median (gr CO2 /gr product)* | gr/week/person | Abs | % | gr/week/person | Abs | % | Abs | % |
| **Beef** | **26.61** | **336** | **8.94** | **1.0** | **100** | **2.66** | **0.4** | **-327.5** | **-70.2** |
| **Pork** | **5.77** | **119** | **0.69** | **0.1** | **50** | **0.29** | **0.0** | **-20.8** | **-58.0** |
| **Chicken** | **3.65** | **168** | **0.61** | **0.1** | **250** | **0.91** | **0.1** | **15.6** | **48.8** |
| Eggs | 3.46 | 140 | 0.48 | 0.1 | 140 | 0.48 | 0.1 | - | - |
| Fish: | 3.49 | 294 | 1.03 | 0.1 | 294 | 1.03 | 0.2 | - | - |
| Milk | 1.29 | 791 | 1.02 | 0.1 | 791 | 1.02 | 0.2 | - | - |
| Cheese | 8.55 | 406 | 3.47 | 0.4 | 406 | 3.47 | 0.6 | - | - |
| **Cereals** | **0.5** | **1204** | **0.60** | **0.1** | **1240** | **0.62** | **0.1** | **0.9** | **3.0** |
| **legumes** | **0.51** | **84** | **0.04** | **0.0** | **250** | **0.13** | **0.0** | **4.4** | **197.6** |
| Vegetables | 0.37 | 1722 | 0.64 | 0.1 | 1722 | 0.64 | 0.1 | - | - |
| Fruits | 0.42 | 1568 | 0.66 | 0.1 | 1568 | 0.66 | 0.1 | - | - |
| **Treenuts** | **1.2** | **21** | **0.03** | **0.0** | **45** | **0.05** | **0.0** | **1.5** | **114.3** |
| **Total** |  | **6853** | **18.2** | **2** |  | **12.0** | **2** | **-325.8** | **-34.3** |

| **South-Islands** | GWP | Baseline Scenario | | | Mediterranean Scenario | | | ΔCO2 (kg/year/person) | |
| --- | --- | --- | --- | --- | --- | --- | --- | --- | --- |
|  |  | Consumption | GHG profile (Kg CO2/year/person) | | Consumption | GHG profile (Kg CO2/year/person) | |  |  |
|  | *Median (gr CO2 /gr product)* | gr/week/person | Abs | % | gr/week/person | Abs | % | Abs | % |
| **Beef** | **26.61** | **245** | **6.52** | **0.7** | **100** | **2.66** | **0.4** | **-201.2** | **-59.2** |
| **Pork** | **5.77** | **119** | **0.69** | **0.1** | **50** | **0.29** | **0.0** | **-20.8** | **-58.0** |
| **Chicken** | **3.65** | **147** | **0.54** | **0.1** | **250** | **0.91** | **0.1** | **19.6** | **70.1** |
| Eggs | 3.46 | 161 | 0.56 | 0.1 | 161 | 0.56 | 0.1 | - | - |
| Fish: | 3.49 | 385 | 1.34 | 0.2 | 385 | 1.34 | 0.2 | - | - |
| Milk | 1.29 | 686 | 0.88 | 0.1 | 686 | 0.88 | 0.1 | - | - |
| Cheese | 8.55 | 427 | 3.65 | 0.4 | 427 | 3.65 | 0.6 | - | - |
| **Cereals** | **0.5** | **1113** | **0.56** | **0.1** | **1240** | **0.62** | **0.1** | **3.3** | **11.4** |
| **legumes** | **0.51** | **98** | **0.05** | **0.0** | **250** | **0.13** | **0.0** | **4.0** | **155.1** |
| Vegetables | 0.37 | 1400 | 0.52 | 0.1 | 1400 | 0.52 | 0.1 | - | - |
| Fruits | 0.42 | 1330 | 0.56 | 0.1 | 1330 | 0.56 | 0.1 | - | - |
| **Treenuts** | **1.2** | **14** | **0.02** | **0.0** | **45** | **0.05** | **0.0** | **1.9** | **221.4** |
| **Totale** |  | **6125** | **15.9** | **2** |  | **12.2** | **2** | **-193.1** | **-23.3** |
